# Excessive daytime sleepiness and gait disturbances in patients with Parkinson’s disease

**DOI:** 10.3389/fnagi.2025.1626247

**Published:** 2025-09-16

**Authors:** Yibo Xie, Maoyun Zhao, Yanjie Guo, Panpan Tian, Sheng Liu, Hongxia Xing

**Affiliations:** ^1^Institute of Physical Education, Xinxiang Medical University, Xinxiang, China; ^2^Key Laboratory of Movement Disorders, The Third Affiliated Hospital of Xinxiang Medical University, Xinxiang, China; ^3^Department of Neurology, The Third Affiliated Hospital of Xinxiang Medical University, Xinxiang, China; ^4^Department of Vascular Surgery, The First Affiliated Hospital of Xinxiang Medical University, Weihui, China; ^5^Institute of Rehabilitation, Xinxiang Medical University, Xinxiang, China

**Keywords:** Parkinson’s disease, excessive daytime sleepiness, gait, gait assessment, wearable sensors

## Abstract

**Background:**

Excessive daytime sleepiness (EDS), which is common in Parkinson’s disease (PD), has been reported to exacerbate gait disturbance in patients with PD, but there is a lack of objective assessment, as well as an unknown specific mechanism. The purpose of our study is to explore the relationship between EDS and gait parameters.

**Methods:**

Sixty-one patients with PD were recruited and divided into the EDS group (*n* = 29) and the non-EDS group (*n* = 32) based on the scores of the Epworth Sleepiness Scale (ESS). The gait metrics of the two groups were then assessed by wearable devices and compared under various walking scenarios.

**Results:**

Compared with the non-EDS group, the EDS group showed significantly shorter step lengths and stride lengths, slower walk speed and gait speed, reduced shank-max forward swing and sagittal angular velocity, and increased phase coordination indices and mean duration of turns. Pearson correlation analysis revealed a significant association between ESS scores and various gait parameters. Furthermore, multiple linear regression analysis confirmed that EDS is an independent factor influencing gait in patients with PD.

**Conclusion:**

EDS was independently associated with gait disturbances in patients with PD, suggesting that EDS symptoms warrant serious attention in clinical practice.

## 1 Introduction

Parkinson’s disease (PD) is a common neurodegenerative disorder that predominantly affects the elderly. It is characterized by motor symptoms, such as bradykinesia, resting tremor, and abnormal gait, and non-motor symptoms, including mood disorders, insomnia, autonomic dysfunctions, and cognitive impairments (Tanner and Ostrem, 2024). Notably, up to 50% of PD patients experience excessive daytime sleepiness (EDS; Abbott et al., 2005), which is often associated with disease progression and dopaminergic drugs, especially dopaminoagonists (Montastruc et al., 2001). It refers to the significant episodes during the day when patients struggle to remain awake and alert, resulting in an uncontrollable need for sleep or inadvertent lapses into sleep (Sateia, 2014). Furthermore, gait disruption is one of the most prevalent motor complaints among PD patients, which can be exacerbated by EDS (Höglund et al., 2015; Chen et al., 2025). Previous studies on the relationship between EDS and gait have used scales to assess gait. Currently, none of the objective research focuses on the relationship between EDS and gait abnormalities. Recent advances in wearable sensor technology have enabled researchers to measure various aspects of gait, including speed and movement patterns (Liu et al., 2022). This makes it possible to objectively assess and record gait impairment in PD patients (Pulliam et al., 2018; Hinchliffe et al., 2024; Borzì et al., 2025).

In this study, a wearable device equipped with inertial sensors was employed to measure the temporal and spatial gait characteristics of PD patients with and without EDS during the Timed Up and Go (TUG) paradigm and 5-meter straight walking paradigm, aiming at investigating the relationship between EDS and gait parameters.

## 2 Materials and methods

### 2.1 Participants

Sixty-one patients with PD were recruited at the outpatient clinic of the Department of Neurology at the Third Affiliated Hospital of Xinxiang Medical University between April 1, 2023, and February 1, 2025. The diagnosis was made according to the MDS criteria for PD (Postuma et al., 2015). The patients with PD included were those at the Hoehn-Yahr (HY) stage ranging from 1 to 2.5. All participants provided written informed consent prior to enrollment in the study. Patients who cannot complete the gait test and those with secondary Parkinsonism syndrome or other superimposed syndromes will be excluded. The study conforms to the ethical guidelines set forth by the Declaration of Helsinki. The study was authorized by the Ethics Committee of the Third Affiliated Hospital of Xinxiang Medical University (approval number K2022-072-01).

### 2.2 Demographic information and clinical evaluations

The demographic data collected included gender, age, height, education, levodopa equivalent daily dose (LEDD), disease duration, past illness, and surgical history. The severity of motor symptoms was evaluated by the MDS-Unified Parkinson’s Disease Rating Scale Part 3 (MDS-UPDRS-III). The severity of EDS was assessed by the Epworth Sleepiness Scale (ESS), ranging from 0 to 24, with higher scores indicating more severe EDS. EDS patients are defined as patients with an ESS score of more than 10 points (Johns, 1991). The psychological conditions and cognitive functions of the patients were assessed by the Hamilton Anxiety Scale (HAMA), Hamilton Depression Scale (HAMD) and Minimum Mental State Examination (MMSE), respectively. The patients’ quality of life (QoL) was evaluated by the Parkinson’s Disease Questionnaire-39 (PDQ-39), and the QoL burden was reflected by the PDQ-39 summary index (PDSI) (Jenkinson et al., 1997). The fatigue level was evaluated by the fatigue severity scale (FSS). The balance was evaluated by the Berg Balance Scale (BBS).

### 2.3 Gait assessments

Gait analysis was performed using the GYENNO MATRIX Wearable Movement and Gait Quantitative Evaluation System, as previously validated (Cai et al., 2023). Sensors are mounted on 10 regions of the body, specifically the chest, waist, left and right wrists, left and right thighs, left and right calves, and left and right feet, and are employed to collect motion data, including trajectories, accelerations, and angular velocities, from these regions, as well as for the detection of the balance index in the walking state: Phase coordination index (PCI; Plotnik et al., 2007; Plotnik et al., 2009). This system is helpful for clinicians to assess movement with objective data. This system has two detecting modes: TUG and 5-meter straight walking. Participants were instructed to walk at a normal speed during the TUG test and at their maximum walking speed for the 5-meter straight walking test, which were used to represent both normal and rapid gait patterns in daily life. These gait measurements have been widely used in previous studies, and presented well efficiency (Gildner et al., 2019; Wang and Zou, 2022). Gait measurements were carried out during the “on-period,” and assessors ensured the safety of participants while they completed the gait tasks.

### 2.4 Statistical analysis

Continuous variables conforming to a normal distribution were presented as mean ± standard deviation (SD), while non-normally distributed variables were characterized by median values accompanied by interquartile range (IQR). Continuous variables were analysed between groups with Student’s t-test for parametric data and Mann–Whitney U test for nonparametric data, determined through distribution normality assessments. Categorical variables were evaluated using chi-square or Fisher’s exact tests. To account for multiple testing, two-sided *p* values were adjusted using the Benjamini-Hochberg (B/H) method to control the false discovery rate (FDR). An association was considered statistically significant if the corresponding B/H-adjusted p value was less than 0.05, indicating an FDR of 5%. The relationship between ESS ratings and gait metrics was examined by Pearson correlation. In the multiple linear regression analysis, the EDS (categorical variable) was set as the independent variable and the gait parameters as the dependent variable, adjusting for the factors of age, sex, disease duration, LEDD and FSS scores. The difference was statistically significant when *p* < 0.05. The statistical analysis was performed with IBM SPSS Statistics 27.0.

## 3 Results

### 3.1 Demographic information and clinical characteristics

Twenty-nine of the 61 patients with PD (47.5%) were classsified into the EDS group. No statistically significant difference was found in age and education years between the two groups. In our study, males were more likely to have EDS which is also in accordance with previous reports (Feng et al., 2021). The patients in the EDS group had a longer duration of the disease, and took more medication than the non-EDS group. Moerover, the MDS-UPDRS-III score, PDSI score, FSS score, and BBS score of patients in the EDS group were significantly higher than those in the non-EDS group (Table 1).

**Table 1:** Baseline participant Clinicodemographic characteristics with different EDS statuses.

Characteristic	Total (*n* = 61)	EDS (*n* = 29)	non-EDS (*n* = 32)	*p* value
Sex (M/F)	33/28	23/6	10/22	<0.001^***^
Age	65.1 ± 8.1	67.1 ± 8.3	63.2 ± 7.6	0.06
Education (years)	8.8 ± 3.3	9.2 ± 2.7	8.5 ± 3.8	0.354
Disease duration (years)	5.2 ± 3.5	6.3 ± 3.5	4.2 ± 3.1	0.016^*^
LEDD (mg)	487.3 ± 207.3	566.7 ± 192.4	415.3 ± 196.2	0.004^**^
MDS-UPDRS-III	39 (27,48)	42 (34,53.5)	31 (23.5,42.8)	0.003^**^
MMSE	26 (24,28)	27 (26,28)	28 (25,29)	0.630
HAMD	6.2 ± 4.7	6.9 ± 4.6	5.6 ± 4.7	0.259
HAMA	8.4 ± 5.3	8.8 ± 5.6	8.1 ± 5.1	0.643
PDSI	0.88 (0.25,1.31)	1.25 (0.44,5.44)	0.75 (0.03,1)	0.006^**^
FSS	36 (15,63)	59 (37,63)	15 (9,38)	<0.001^***^
BBS	51 (47,55)	49 (45,51)	54 (51,56)	<0.001^***^

Data are shown as mean ±standard deviation or median (P25, P75). EDS Patients with EDS, non-EDS Patients without EDS, LEDD, Levodopa Equivalent Daily Dose; MDS-UPDRS-III, Movement Disorders Society Unified Parkinson’s Disease Rating Scale part 3; MMSE, Brief Mental State Examination; HAMA, The Hamilton Anxiety Scale; HAMD, Hamilton Depression Scale; PDSI, Parkinson’s disease summary index; FSS, fatigue severity scale; BBS, Berg Balance Scale. *p < 0.05, **p < 0.01, ***p < 0.001.

### 3.2 Gait parameters in different walking paradigms

**Figure 1:**
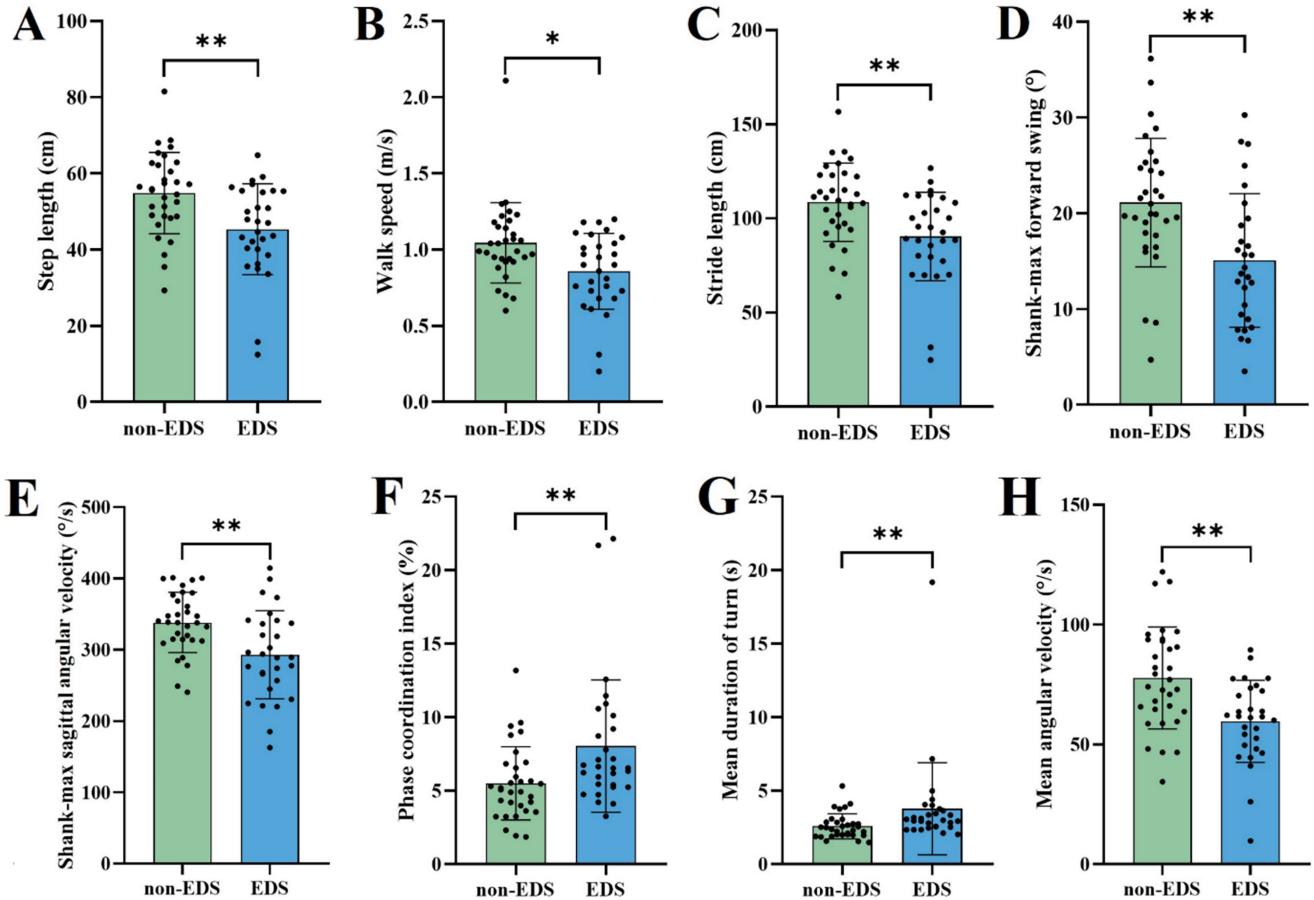
Comparison of gait parameters with significant differences in the TUG between different EDS states. EDS Patients with EDS, non-EDS Patients without EDS. *p < 0.05, **p < 0.01, ***p < 0.001. Multiple comparison correction was performed using Benjamini/Hochberg (BH).

In the task of TUG, the patients in the EDS group had shorter step length and stride length, slower walk speed and more reduced shank-max forward swing, shank-max sagittal angular velocity and mean angular velocity, compared to non-EDS group (Table 2 and Figure 1).

**Table 2:** Comparison of gait parameters with different EDS statuses in the TUG.

Characteristic	Total (*n* = 61)	EDS (*n* = 29)	non-EDS (*n* = 32)	*p* value
Step length (cm)	50.3 ± 12.2	45.3 ± 12.0	54.9 ± 10.7	0.005^**^
Walk speed (m/s)	0.97 (0.78,1.11)	0.90 (0.71,1.06)	1.01 (0.93,1.17)	0.026^*^
Stride length (cm)	102.90 (86.89,114.42)	92.62 (78.42,109.65)	110.20 (96.02,123.04)	0.005^**^
stride duration (s)	1.08 ± 0.11	1.08 ± 0.11	1.08 ± 0.11	0.904
Cadence (steps/min)	113.44 ± 11.84	113.92 ± 11.84	113.01 ± 11.06	0.875
Double support (%)	19.47 (16.95,23.46)	19.03 (16.38,23.11)	20.13 (17.61,22.91)	0.604
Stance (%)	59.32 (57.71,61.04)	58.74 (57.59,61.02)	59.46 (58.33,61.29)	0.604
Swing (%)	40.68 (38.96,42.29)	41.27 (38.98,42.41)	40.54 (38.71,41.67)	0.604
Shank-max forward swing (°)	18.29 ± 7.43	15.07 ± 6.96	21.11 ± 6.71	0.004^**^
Shank-max backward swing (°)	45.13 (42.87,47.40)	44.34 (40.62,46.02)	45.99 (43.75,47.76)	0.052
Shank-max sagittal angular velocity (°/s)	316.66 ± 56.75	292.92 ± 56.75	338.17 ± 42.18	0.004^**^
Stride velocity difference (m/s)	0.049 ± 0.018	0.048 ± 0.013	0.050 ± 0.021	0.847
Mean phase difference (%)	2.99 (2.37,4.12)	3.25 (2.57,4.52)	2.92 (1.97,3.67)	0.904
Phase coordination index (%)	5.65 (4.45,7.70)	6.53 (5.32,9.66)	5.12 (3.71,6.75)	0.009^**^
Mean duration of turn (s)	2.73 (2.20,3.34)	2.99 (2.53,3.69)	2.42 (1.97,2.84)	0.004^**^
Mean angular velocity (°/s)	69.10 ± 21.30	59.60 ± 17.14	77.71 ± 21.26	0.004^**^

Data are shown as mean ± standard deviation, median (P25, P75). *p < 0.05, **p < 0.01, ***p < 0.001. EDS, Patients with EDS, non-EDS Patients without EDS. Multiple comparison correction was performed using Benjamini/Hochberg (BH), the *p* value is the adjusted *p* value based on BH.

**Figure 2:**
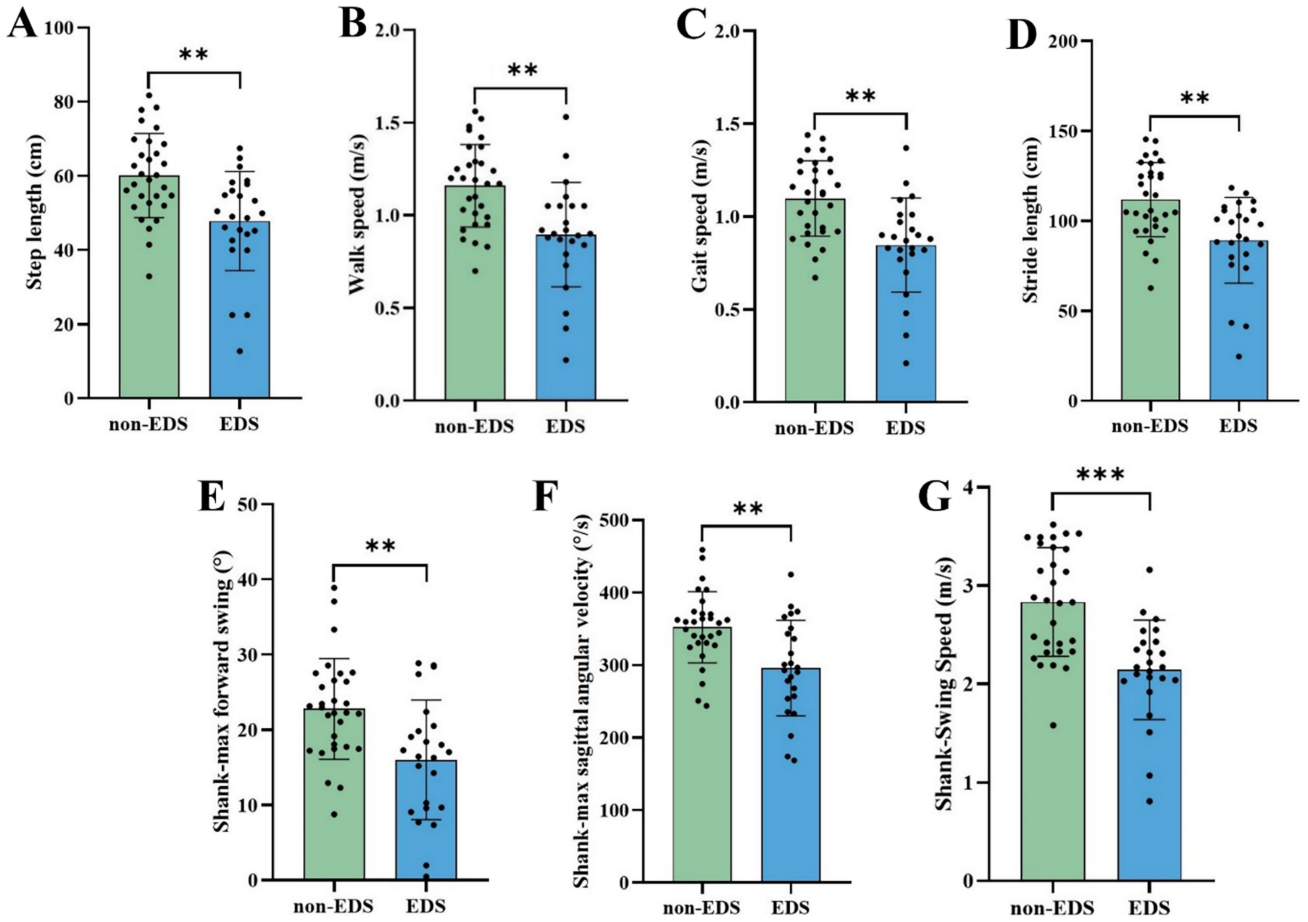
Comparison of gait parameters with signific**ant** differences in the 5-meter straight walking between different EDS states. EDS Patients with EDS, non-EDS Patients without EDS. *p < 0.05, **p < 0.01, ***p < 0.001. Multiple comparison correction was performed using Benjamini/Hochberg (BH).

In the 5-meter straight walking, EDS group had shorter step length, stride length, smaller shank-max forward swing, shank-max sagittal angular velocity and shank—swing speed compared to non-EDS group, consistent with the TUG paradigm (Table 3 and Figure 2).

**Table 3:** Comparison of gait parameters with different EDS statuses in the 5-meter straight walking.

Characteristic	Total (*n* = 54)	EDS (*n* = 24)	non-EDS (*n* = 30)	*p* value
Step length (cm)	54.74 (47.46,63.55)	49.46 (43.04,57.32)	59.56 (52.53,68.76)	0.002^**^
Walk speed (m/s)	1.04 ± 0.28	0.90 ± 0.28	1.16 ± 0.22	0.001^**^
Gait speed (m/s)	0.99 ± 0.26	0.85 ± 0.25	1.10 ± 0.20	0.001^**^
Stride length (cm)	103.16 (88.64,118.91)	94.87 (80.28,105.85)	114.37 (97.90,129.34)	0.002^**^
Stride duration (s)	1.06 ± 0.11	1.09 ± 0.13	1.04 ± 0.09	0.204
Cadence (step/min)	115.73 ± 12.72	113.63 ± 15.46	117.40 ± 9.99	0.369
Double support (%)	20.60 (18.48,23.55)	21.39 (16.72,25.15)	20.60 (18.51,23.00)	0.903
Swing (%)	40.16 (38.11,41.24)	40.19 (37.24,42.51)	40.16 (38.57,41.11)	0.903
Stance (%)	59.84 (58.77,61.89)	59.81 (57.49,62.76)	59.84 (58.89,61.43)	0.903
Shank-max forward swing (°)	19.77 ± 7.98	15.99 ± 7.95	22.79 ± 6.71	0.002^**^
Shank-max backward swing (°)	46.10 (43.03,48.31)	44.17 (42.15,47.66)	46.66 (44.27,48.84)	0.114
Shank-max sagittal angular velocity (°/s)	327.17 ± 63.25	295.86 ± 65.94	352.23 ± 49.02	0.002^**^
Shank-Swing Speed (m/s)	2.42 (2.16,3.06)	2.17 (2.03,2.43)	2.84 (2.33,3.40)	<0.001^***^

Data are shown as mean ± standard deviation, median (P25, P75). *p < 0.05, **p < 0.01, ***p < 0.001. EDS Patients with EDS, non-EDS Patients without EDS. Multiple comparison correction was performed using Benjamini/Hochberg (BH), the *p* value is the adjusted P value based on BH.

### 3.3 The correlation of EDS with gait parameters

There was a significant correlation between the TUG gait parameters and the 5-meter straight walking parameters and ESS scores in PD patients. In TUG, ESS scores were negatively correlated with step length (r = −0.26, *p* < 0.05), stride length (r = −0.26, *p* < 0.05), shank-max forward swing (r = −0.341, *p* < 0.01), shank-max sagittal angular velocity (r = −0.273, *p* < 0.05), and mean angular velocity (r = −0.358, *p* < 0.01) and positively correlated with phase coordination index (r = 0.253, *p* < 0.05). In 5-meter straight walking, the ESS scores were also associated with step length (r = −0.319, *p* < 0.05), walk speed (r = −0.355, *p* < 0. 01), gait speed (r = −0.375, *p* < 0.01), stride length (r = −0.326, *p* < 0. 05), shank-max forward swing (r = −0.331, *p* < 0.05), shank-max sagittal angular velocity (r = −0.353, *p* < 0.01), and shank-swing speed (r = −0.431, *p* < 0.01).

### 3.4 The multiple linear regression analysis of EDS and gait parameters

To investigate the relationship between EDS and individual gait parameters and control for the effects of confounding factors (including age, sex, disease duration, LEDD, and FSS score), multiple linear regression analysis were performed in this study. The unadjusted crude model showed that EDS had a significant effect on step length (B = −9.507, *p* = 0.002), walk speed (B = −0.188, *p* = 0.006) and stride length (B = −18.201, *p* = 0.002)in TUG, and step length (B = −12.266, *p* < 0.001), walk speed (B = −0.265, *p* < 0.001), and stride length (B = −22.681, *p* < 0.001) in the 5-meter straight walking, were all significant. Furthermore, after adjusting for age, disease duration, gender, LEDD and FSS score, EDS still had a significant effect on step length (B = −10.417, *p* = 0.009), walk speed (B = −0.232, *p* = 0.01) and stride length (B = −20.162, *p* = 0.01) in TUG, and step length (B = −12.195, *p* = 0.007), walk speed (B = −0.219, *p* = 0.018) and stride length (*B* = −21.858, *p* = 0.008) in the 5-meter straight walking, remained significant (Table 4).

**Table 4:** Multiple linear regression analysis of EDS and gait parameters before and after model adjustment.

Gait parameters	unadjusted		Adjusted			
	*B(95%CI)*	β	*p* value	*B(95%CI)*	β	*p* value
Step length^1^	−9.507 (−15.306 ~ −3.709)	−0.393	0.002**	−10.417 (−18.166 ~ −2.667)	−0.430	0.009^**^
Walk speed^1^	−0.188 (−0.320 ~ −0.056)	−0.349	0.006**	−0.232 (−0.407 ~ −0.058)	−0.431	0.01^*^
Stride length^1^	−18.201 (−29.537 ~ −6.864)	−0.386	0.002**	−20.162 (−35.285 ~ −5.039)	−0.427	0.01^*^
Step length^2^	−12.266 (−19.029 ~ −5.504)	−0.451	<0.001^***^	−12.195 (−20.828 ~ −3.562)	−0.448	0.007^**^
Walk speed^2^	−0.265 (−0.403 ~ −0.128)	−0.473	<0.001^***^	−0.219 (−0.399 ~ −0.040)	−0.391	0.018^*^
Stride length^2^	−22.681 (−34.841 ~ −10.521)	−0.461	<0.001^***^	−21.858 (−37.661 ~ −6.055)	−0.444	0.008^**^

B regression coefficient, CI confidence interval, β standardized regression coefficient. *p < 0.05, **p < 0.01, ***p < 0.001.

1 TUG.

2 5-meter straight walking.

## 4 Discussion

In this study, nearly half of the patients with PD exhibited EDS, and male patients and those with a longer disease duration were more likely to present with EDS. These results are consistent with previous reports (Mengdie et al., 2022; Chahine et al., 2017; Liu et al., 2021). The LEDD of patients in the EDS group was significantly higher than patients in the non-EDS group, which is common in reports related to EDS. EDS is more common in PD patients taking higher doses of Dopaminoagonists and levodopa (Liu et al., 2022). Even most antiparkinsonian medications (Koller et al., 2005; Hauser et al., 2014) can induce or aggravate EDS due to their sedative effects (Arnulf and Leu-Semenescu, 2009). However, some drugs can also improve EDS, such as piribedil (Eggert et al., 2014) and selegiline (Gallazzi et al., 2021). Therefore, it is very important to carefully assess EDS and select reasonable drugs for patients.

Longitudinal studies on PD have identified several risk factors for EDS, including age, gender, and disease duration (Zhu et al., 2016; Tholfsen et al., 2015). Additionally, we found that there was a significant difference in FSS scores between the two groups at baseline. So we adjusted these risk factors that might affect the outcome. By quantifying gait parameters more objectively, we found that PD patients with EDS exhibited more severe gait impairment. Specifically, EDS may be associated with the deterioration of both normal walking gait and fast walking gait in PD patients. Even the effect of EDS on gait remained significant after adjusting for confounders such as sex, age, disease duration, LEDD and FSS. The results are consistent with previous scale-only studies showing that EDS is associated with a wider range of motor and nonmotor PD features including axial/postural/gait deficits, depression, and pain (Höglund et al., 2015). One reason may be that EDS is often accompanied by cognitive impairments such as poor concentration (Bohnen et al., 2012), memory loss and executive dysfunction (Gasa et al., 2013). This cognitive dysfunction affects the patient’s gait in the early stages of PD (Rochester et al., 2017). During walking, cognitive functions play an important role in gait planning, maintenance of balance, and perception of and response to the environment. Impaired cognitive function may lead to gait abnormalities such as disorientation and delayed reaction time when walking, increasing the risk of falls. The observed sensory integration delays may arise from attentional deficits associated with sleep disturbances. Effective postural control fundamentally relies on the central nervous system’s capacity to synchronize visual cues, vestibular signals, and proprioceptive feedback in real-time (Teasdale and Simoneau, 2001; Ouchi et al., 1999); this sensory integration requires a high degree of attention, especially as the efficiency of sensory inputs decreases with age, which may affect gait performance (Tyagi et al., 2017). Although previous studies have attributed EDS-related gait deficiency to impaired attention or executive function, we found comparable MMSE scores between groups, which seems contradictory. However, the MMSE primarily assesses general cognition and lacks sensitivity to executive dysfunction in specific domains (Hausdorff, 2005). In PD patients, gait control relies greatly on prefrontal-mediated processes that cannot be captured by the MMSE.

Step length shortening is consistent with the “sequence effect” of PD, in which there is a gradual decay in amplitude of movement, which is usually associated with basal ganglia dysfunction. EDS may exacerbate this phenotype through nigrostriatal dopamine depletion, as animal models show that sleep deprivation accelerates the loss of dopaminergic neurons (Parhizkar et al., 2023).

Importantly, gait disturbances caused by EDS are a direct threat to patient safety and quality of life. Both gait speed and stride length, which are predictors of falls in older adults (Kyrdalen et al., 2019), are significantly reduced in PD patients with EDS, who are at very high risk of falling (Fasano et al., 2017; Allen et al., 2013). Falls frequently lead to fractures (Kalilani et al., 2016), hospitalization (Paul et al., 2017), functional decline, significantly reducing patients’ independence and quality of life (Rascol et al., 2015). Therefore, early detection of EDS provides a critical window for intervention to mitigate future gait deterioration and fall risk. We recommend emphasizing the management of EDS in early PD, including nonpharmacological therapies such as repetitive transcranial magnetic stimulation that may improve both Sleep problems and motor function (Zhang et al., 2022).

While the use of scales and direct observation by clinicians is still common in routine assessments, a growing number of studies has demonstrated the added value of wearable inertial sensors for objective gait analysis in patients with PD (Pulliam et al., 2018; Ricci et al., 2020; Isaacson et al., 2019; Dai et al., 2021; Perez-Ibarra et al., 2020; Rigas et al., 2012; Demrozi et al., 2020; Mariani et al., 2013). On this basis, we used the Wearable Movement and Gait Quantitative Assessment System to obtain accurate quantitative gait parameters during the TUG and the 5-meter straight walking task. We aimed to provide an objective and intuitive assessment of how EDS affects gait function in patients with PD.

There are certain restrictions on this study. The sample size of the study was relatively small. Consequently, the reliability and generalizability of the results may be limited. To enhance the statistical significance of the findings, future research should consider increasing the sample size. Using only the MMSE as a cognitive assessment tool is insufficient, and future research could incorporate more tests, especially for specific cognitive functions such as attention and integration. This study did not analyse patients for the specific type of medication they were using and only focused on patients with PD in the early stages of the disease, future studies should include detailed medications and patients in all periods of time. Additionally, due to the cross-sectional nature of this study, it was not possible to establish a causal relationship between gait impairment and EDS. Future investigations could adopt a longitudinal study design to better understand the long-term effects of EDS on gait function by tracking changes over time.

## Data Availability

The raw data supporting the conclusions of this article will be made available by the authors, without undue reservation.
